# Exploring Socio‐Demographic Characteristics of Caregivers Who Indicated a Child Was Substantially Affected by Others' Drinking in Australia

**DOI:** 10.1111/dar.14087

**Published:** 2025-05-27

**Authors:** Cassandra Hopkins, Sandra Kuntsche, Robyn Dwyer, Heng Jiang, Anne‐Marie Laslett

**Affiliations:** ^1^ Centre for Alcohol Policy Research La Trobe University Melbourne Australia; ^2^ Bouverie Centre La Trobe University Melbourne Australia; ^3^ Department of Public Health La Trobe University Melbourne Australia; ^4^ Melbourne School of Population and Global Health The University of Melbourne Melbourne Australia; ^5^ Care Economy Research Institute La Trobe University Melbourne Australia

**Keywords:** alcohol, children, harm to others, social determinants of health

## Abstract

**Introduction:**

Children's health and wellbeing is influenced by the social, economic and environmental conditions in which they live, known as the social determinants of health (SDH). This study examines caregivers' socio‐demographic characteristics and reports of children substantially affected by others' drinking (i.e., caregivers' interpretations of severity).

**Methods:**

A sample of 705 adults living with children under 18 years from the 2021 Australian Alcohol's Harm to Others study indicated how much a child was negatively affected by others' drinking (excluding their own) in the past year. Responses were categorised as ‘substantially affected’ or ‘less substantially affected’. Logistic regressions examined associations of socio‐demographic characteristics and reports of substantially affected children.

**Results:**

Amongst caregivers, 5.4% indicated a child was substantially affected by others' drinking in the past year. Identifying as a woman (95% CI 1.17–5.13, *p* = 0.017), experiencing financial stress (95% CI 2.01–7.70, *p* = 0.000), being a single caregiver (95% CI 1.35–6.56, *p* = 0.007) or living in an area with a lower Socio‐Economic Indexes for Areas score (95% CI 1.03–3.94, *p* = 0.039) was associated with an increased likelihood of indicating a child was substantially affected by others' drinking.

**Discussion and Conclusions:**

Our findings highlight that caregiver social disadvantage is associated with indicating a child was substantially affected by others' drinking. Policies addressing alcohol consumption and social disadvantage affecting children's wellbeing are needed, particularly financial support for families experiencing financial stress and single caregivers. Larger and more targeted studies are needed to further examine SDH and outcomes for children related to others' drinking.

## 1 Introduction

Children's health and wellbeing is influenced by the social, economic and environmental conditions in which they live, also known as the social determinants of health (SDH). These determinants also include broader forces and systems, such as political, education or healthcare systems, that influence those conditions [1, 2]. Health and wellbeing outcomes follow a social gradient, where those in more disadvantaged socio‐economic circumstances experience poorer health outcomes, compared to those in advantaged circumstances [3]. In regard to children, disadvantages experienced by children's caregivers and families have an impact on children's own health and wellbeing, where inequalities present early in life can persist into adulthood [4, 5]. Research has found that an individual's drinking may negatively affect family and household members, including children [6, 7]. Consequences of children's experiences of harm from others' drinking has been shown to extend beyond childhood, into adulthood, contributing to mental and physical health issues, and difficulties with trust and relationships [8, 9]. While there is some information on varying impacts of others' drinking on children globally [10, 11, 12], there is limited literature exploring the role of social disadvantage in shaping this broader range of alcohol‐related effects on children in the general population. Therefore, understanding and addressing the implications of SDH in relation to the impact of others' drinking on children is important given, the potential for health and social disadvantages to exacerbate negative effects children's wellbeing.

Previous Australian research has found that 22% of caregivers indicated that a child had experienced harm from others' drinking [12], while in New Zealand this figure was 17% [13] and in the United States, it was 7.4% [14]. Harms measured in previous studies have often focused on ‘any harm’ to children, with few measuring and reporting on the severity of harm to children. An Australian study of caregivers that did consider severity found that when asked ‘How much has the drinking of other people negatively affected your children/the children you are responsible for?’, 3% of respondents indicated ‘a lot’ [12]. Similarly, few studies have examined relationships between SDH and more substantial harms to children from others' drinking, and available findings are mixed. Physical harms and exposure to violence are commonly considered more serious forms of harm to children [15]. A cross‐national study on children's experience of these forms of harm from others' drinking found that having higher education (a commonly used proxy for SES [16]) did not mitigate direct physical harms or exposure to violence amongst children [17]. Conversely, a review of reviews focusing on the intersections of alcohol and other drug ‘misuse’ (not clearly defined), intimate partner violence and ‘child abuse’ found that amongst families experiencing poverty and housing instability, the likelihood of these problems (alcohol and other drug consumption, intimate partner violence and child abuse) increased [18].

While researchers have paid minimal attention to SDH in harms to children from others' drinking, broader literature on child maltreatment, neglect and abuse may inform understandings of the relationships between SDH and adverse outcomes for children [19]. A systematic review found that poverty, low parental education attainment, housing instability and food insecurity were associated with child maltreatment [20]. The authors found that poverty was the SDH most strongly and consistently associated with maltreatment, and this relation held at country, community, household and individual levels of measuring poverty. Children in families experiencing poverty are over‐represented in child protection interventions [21], and this relation is exacerbated where problematic alcohol and other drug consumption is present [22]. A study on substantiated cases within the Child Protection System in Victoria, Australia, from 2001 to 2005 found that caregivers' alcohol consumption was a predictor of more intensive official responses such as protective interventions and court orders, and in cases where alcohol consumption was recorded, caregivers were more likely to be socially and economically disadvantaged, as measured by living in public housing and having lower incomes [23]. However, it is important to note that the over‐representation of these caregivers and families in child protection systems may be a function of over‐surveillance such that they are more likely to come into contact with government‐funded social and economic support systems [24, 25].

Given the established associations between SDH and child maltreatment in general, this study examines relations between SDH and harm to children from others' drinking – a specific type of harm within the broad spectrum of child maltreatment. The study analyses caregiver perceptions of ‘how much’ a child was negatively affected by someone else's drinking, where responses have been categorised as ‘substantially affected’ versus ‘less substantially affected’. This takes a broader approach that considers caregivers' subjective perceptions of negative effects, capturing a spectrum of potential negative effects beyond (and including) severe maltreatment. Examining how children are substantially affected by others' drinking in the contexts of SDH is important for understanding the complex effects of alcohol use on children's wellbeing. While the proportion of children substantially affected by the drinking of others may be low in general population surveys, the consequences of being substantially affected may have severe and long‐term implications that may extend into adulthood. Identifying the social and economic factors associated with substantial harm from others' drinking to children may broaden understanding of the needs of families. This may assist in identifying appropriate policy responses that can support children's wellbeing in the context of their broader social environments [26]. This analysis examines the association between socio‐demographic characteristics (individual, household and area‐based) of caregivers who live with children and their perceptions of how much a child was affected by someone else's drinking in the past 12 months.

## 2 Methods

### 2.1 Study Design and Sample Description

The analysis used data from the 2021 Australian Alcohol's Harm to Others survey, a national sample of 2574 adults (see [27] for a description of the overall sample), combining two survey methods: random digit dialling of Australian mobile phones and the Life in Australia panel survey [28]. The survey was conducted in November and December 2021. The response rate for the random digit dialling survey was 5.5% and the cumulative response rate for the Life in Australia panel survey was 6.1%. The data are self‐reported by the respondent and include information on the impact of others' drinking on the respondent and on children for whom the respondent is a carer. The current analyses are based on a subgroup of 723 adult respondents (see Table 1) who indicated they lived with at least one child aged ≤ 17 years. The survey was approved by La Trobe University's Human Ethics Research Committee (HEC20518).

**TABLE 1:** Sample characteristics of caregivers who live with at least one child under 18 years, raw numbers (*n*) and unweighted percentages (%).

Characteristics	*n*	%
Gender^a^ (*n* = 702)		
Men	320	45.6
Women	382	54.4
Education (*n* = 705)		
Bachelor's degree or higher	373	52.9
Less than a bachelor's degree	332	47.1
Financial stress (*n* = 701)		
No	571	81.5
Yes	130	18.5
Household structure (*n* = 704)		
Two or more adults	632	89.8
Single adult	72	10.2
Type of household (*n* = 691)		
Owned outright or with mortgage	496	71.8
Renting/do not own home	195	28.2
People per bedroom^b^ (*n* = 694)		
Two or less	675	97.3
More than two	19	2.7
SEIFA (*n* = 695)		
Middle to high SEIFA	483	69.5
Low SEIFA	212	30.5

*Note*: Small numbers of respondents did not answer socio‐demographic and consequently numbers do not total 705.

Abbreviation: SEIFA, Socio‐Economic Indexes for Areas.

^a^ Excludes responses of ‘non‐binary’ (due to small numbers).

^b^ Overcrowding is defined as more than two people per bedroom (see [28, 29]).

#### 2.1.1 Outcome Variable

The 2021 Australian Alcohol's Harm to Others survey asked respondents about a range of harms from someone else's drinking that either the respondent or children they care for had experienced. The outcome measure used in this analysis is a measure of caregivers' subjective perceptions about ‘how much’ a child in their care was ‘negatively affected’ by someone else's drinking. Importantly, this question was asked after caregivers had been asked six specific ‘harm’ questions. These were whether a child in their care had been: verbally abused; physically harmed; left unsupervised; financially harmed; witness to serious violence; or the subject of a child protection call. This sequencing likely influenced caregivers' interpretations of what it meant for a child to be ‘negatively affected’. However, it is also possible that caregivers were rating other potential negative effects that were not asked about in the survey.

Moreover, caregivers may have interpreted the question of ‘how much’ as either (or both) the frequency or the severity of negative effects experienced by the child/children in their care. To address some of this ambiguity, the analysis incorporated an additional measure that asked the same question (How much has the drinking of other people negatively affected these children in the last twelve months?) yet required caregivers to rate the impact on a scale from 1 (a little) to 10 (a lot). The outcome variable in this analysis is therefore a subjective measure of the impact or severity of ‘how much’ a child was negatively affected by someone else's drinking, as perceived by their caregivers. Responses were categorised into two groups: ‘substantially affected’ or ‘less substantially affected’, to distinguish between higher and lower levels of reported negative effects.

Caregivers were asked (Q1) to indicate how much (‘a lot’ [1], ‘a little’ [2] or ‘not at all’ [3]) the drinking of other people (excluding the respondent) had negatively affected the children in the last 12 months. Respondents who did not provide an answer (*n* = 18) were excluded from the analyses, resulting in 705 valid responses. Caregivers who indicated a child was affected either ‘a lot’ or ‘a little’ were further asked (Q2) to specify on a scale of 1–10 (with 10 being more substantial): *How much has the drinking of other people negatively affected these children in the last twelve months*? A binary variable was created where answers of less than 5 were coded as ‘less substantial harm’ [0] and answers of 5 and above were coded as ‘substantial harm’ [1].

To generate the final outcome variable used in the analysis, a binary variable (*n* = 705) was created by combining answers of ‘a lot’ (from Q1 above) and ‘substantial harm’ (from Q2 above) to identify those who indicated a child had been substantially affected (1) from those who indicated children were affected ‘a little’, less substantially or not at all (0).

#### 2.1.2 Predictor Variables

The analysis uses individual, household and area‐based socio‐demographic characteristics of caregivers. Due to substantial missing data, individual and household income variables were not included. Instead, caregivers' educational attainment and recent experiences of financial stress were used as indicators of financial disadvantage. Non‐responses were excluded pairwise to maintain statistical power.

### 2.2 Individual‐Level Socio‐Demographic Characteristics

Respondents were asked ‘How would you describe your gender?’ and were coded as men (0), women (1) or non‐binary (2). Due to small numbers, respondents answering ‘non‐binary’ or not answering (*n* = 3) were excluded. Participants were asked ‘What is the highest level of education or training that you have completed?’ and coded as Bachelor's degree or higher (0) and lower than a Bachelor's degree (1). The financial stress measure comprised responses to three questions (adapted from the Financial Resources Study [29]) about respondents' experiences in the past year: (i) could you have raised $2000 within a week for an emergency; (ii) have you sought financial help from family or friends; (iii) have you sought assistance from welfare or community organisations. Participants who experienced at least one financial stress item (1) were distinguished from those not indicating they experienced any financial stress items (0). Four respondents who had missing data on all three financial stress items were excluded from the analysis (*n* = 701).

### 2.3 Household‐Level Socio‐Demographic Characteristics

Household structure was determined by the number of adults in the household and coded as single adult (1) and two or more adults (0) with one non‐response excluded. Participants were also asked: How many adults 18 years or older, and how many children aged 0–12 and 13–17 usually live in their household (two non‐responses); and How many bedrooms are in their household (nine non‐responses). The total number of people (adults and children) living in the household was divided by the number of bedrooms in the household and coded as two people or less per bedroom (0) and more than two people per bedroom (1) as a measure of overcrowding [30, 31]; 11 non‐responses were excluded. Participants indicated whether they lived in and owned their house outright or did so with a mortgage (0) or whether they were renting or did not own the household they lived in (1), with 14 non‐responses excluded.

### 2.4 Area‐Level Socio‐Demographic Characteristics

Participants were asked to provide their postcode in the survey (10 non‐responses). Postcodes were used to assign Socio‐Economic Indexes for Areas (SEIFA) to rank participants according to relative socio‐economic disadvantage quintile. SEIFA is based on Census data from 2021 [32]. The SEIFA variable was then coded as ‘middle to high SEIFA’ (0) and ‘low SEIFA’ (1).

### 2.5 Analysis

Data were analysed using Stata 18.0 [33]. Descriptive statistics are presented to show the general prevalence of children substantially affected by others' drinking and socio‐demographic factors. Non‐responses were excluded through a pairwise approach to maintain statistical power in the analysis. Multiple bivariable logistic regressions were used to analyse the relationships between the socio‐demographic characteristics of caregivers and indicating substantial harm to children. Multiple bivariable logistic regressions were used due to low counts of the outcome variable and to maintain statistical power.

## 3 Results

### 3.1 Sample Characteristics

Table 1 shows characteristics of the 705 caregivers who indicated they lived with one or more children. Men represented 45.6% of the sample. Over half (52.9%) of the caregivers held a Bachelor's degree or higher. Almost one‐fifth (18.5%) of caregivers indicated they had experienced financial stress in the past 12 months. Around 10% of participants indicated they were living in single adult households with children. Over a quarter (28.2%) of participants were renting or living in a residence they did not own. Around 30% of caregivers were living in areas ranked as low SEIFA.

### 3.2 Children Substantially Affected From Someone Else's Drinking

Table 2 shows the percentage of caregivers living with children who indicated a child was not at all affected (88.2%), negatively affected ‘a little’ (7.9%) and negatively affected ‘a lot’ (3.8%). Amongst the caregivers who indicated ‘a little’ or ‘a lot’ of harm, when asked to indicate on a scale of 1–10 how much a child was affected by the drinking of other people, 57.5% indicated it was less substantial, and 42.5% indicated it was substantial. The combined outcome measure (see Section 2) shows that 5.4% of caregivers indicated that one or more children in their care had been substantially affected by someone else's drinking in the past year.

**TABLE 2:** Percentages of caregivers who live with at least one child under 18 years and how much a child was affected by someone else's drinking, raw numbers (*n*) and unweighted percentages (%).

Variable	*n*	%
How much has the drinking of others affected a child (*n* = 705)		
Not at all	622	88.2
A little	56	7.9
A lot	27	3.8
Scale of how much a child had been affected (scale 1–10; *n* = 80^a^)		
Less substantially affected	46	57.5
Substantially affected	34	42.5
Substantial harm		
No	677	94.6
Yes	38	5.4

^a^Only participants who indicated ‘a lot’ or ‘a little’ were asked to indicate on a scale of 1–10 how much had a child been affected by others' drinking.

### 3.3 Socio‐Demographic Characteristics of Caregivers and Indicating a Child Was Substantially Affected by Others' Drinking

Table 3 shows the likelihoods of caregivers indicating a child had been substantially affected by someone else's drinking in the past 12 months according to various socio‐demographic characteristics of caregivers. The logistic regression analyses show that women were significantly more likely than men to indicate a child was substantially affected because of someone else's drinking (odds ratio [OR] 2.45, confidence interval [CI] 1.17–5.13, *p* < 0.017). Caregivers experiencing financial stress compared to those who did not were more likely to indicate a child was substantially affected by someone else's drinking (OR 3.94, CI 2.01–7.70, *p* < 0.000). Household structure (single vs. two or more adults) was also significant, with single caregivers more likely to indicate a child was substantially affected by someone else's drinking (OR 2.97, CI 1.35–6.56, *p* < 0.007). Caregivers living in low SEIFAs were more likely to indicate a child was substantially affected compared to caregivers living in middle to high quintiles (OR 2.02, CI 1.03–3.94, *p* < 0.039).

**TABLE 3:** Odds ratios of caregivers indicating a child had been substantially affected by someone else's drinking in the past 12 months.

Variable	Odds ratio	95% CI	*p*
Gender			
Men	Ref		
Women	2.45	1.17–5.13	0.017*
Education			
Bachelor's degree or higher	Ref		
Less than a bachelor's degree	1.01	0.53–1.95	0.972
Financial stress			
No	Ref		
Yes	3.94	2.01–7.70	0.000***
Household structure			
Two or more adults	Ref		
Single adult	2.97	1.35–6.56	0.007**
Type of household			
Owned outright or with mortgage	Ref		
Renting/do not own home	1.59	0.80–3.16	0.185
People per bedroom			
Two or less	Ref		
More than two	3.53	0.98–12.72	0.053
SEIFA			
Middle to high SEIFA	Ref		
Low SEIFA	2.02	1.03–3.94	0.039*

*Note*: Total *n* in bivariate logistic regression varied from 691 to 705. Asterisks indicate statistical significance: *p < 0.05; **p < 0.01; ***p < 0.001.

Abbreviations: CI, confidence interval; SEIFA, Socio‐Economic Indexes for Areas.

## 4 Discussion

Within our sample of Australian caregivers, 5.4% indicated a child had been substantially affected by someone else's drinking in the past 12 months. We describe for the first time in Australia, that identifying as a woman, experiencing financial stress, living in a single adult household or living in a disadvantaged socio‐economic area was associated with an increased likelihood of indicating a child had been substantially affected by someone else's drinking. While few studies have explored the relationship between SDH and children's experience of harm from another person's drinking, our findings are consistent with the broader literature on alcohol‐related harms and social and economic disparities [34, 35]. This analysis provides an additional perspective on the effects that children may experience because of others' drinking that contributes to and extends the existing research by examining the circumstances that increase the likelihood of being substantially affected.

Data used in this analysis were collected in 2021. During this year in Australia, financial stress was relatively high – most likely due to the impacts of the COVID‐19 pandemic [36]. Our study found that 18.6% of caregivers indicated they had experienced at least one financial stress item in the past 12 months, which was consistent with other national surveys on families with children, conducted at a similar time [36]. Financial stress in families may contribute to tensions or conflict within the home [37], which may lead to increased alcohol consumption [35] and pressures on the mental wellbeing of parents and caregivers [36], due to compounding stressors. Additionally, financial stress may exacerbate existing conflict or strains within the household, which may contribute to children's experience of harm from others' drinking. Our finding is consistent with the child maltreatment literature, where children living in families that are experiencing financial stress are at an increased likelihood of experiencing maltreatment [38]. In light of our finding of associations between caregivers' experiences of financial stress and increased likelihood of indicating a child was substantially affected because of others' drinking, it is of concern that financial stress amongst caregivers in Australia may have increased due to increasing costs of living and inflation [39]. In the present analysis, information on socio‐demographic characteristics and socio‐economic circumstances of the person whose drinking has affected the child was not available. Future alcohol's harm to others studies should consider including more detail on the person whose drinking causes harm to children as it may assist in explaining some of the associations found in our study.

The present analysis found that single caregivers were more likely to indicate a child was substantially affected by someone else's drinking than households with two or more adult caregivers. Being a single caregiver can be considered a SDH due to the social and economic challenges it presents, which can impact both caregiver and child wellbeing [40]. Additionally, most single caregivers in Australia are women [41] who may face additional social and economic challenges because of a combination of gender inequalities, caregiving responsibilities and structural barriers. Single caregivers may be more likely to indicate their child was substantially affected by someone else's drinking because they may have less economic or social support than dual caregiver households. As a consequence, single caregivers may rely on informal caregiving arrangements, which may expose their children to situations involving heavy alcohol use while the caregiver was not present [42]. A single caregiver's capacity for supervision may also be stretched with the presence of other adults, particularly in situations of heavy alcohol consumption, which may increase the level of exposure of the child to other adults' drinking. Additionally, neighbourhood disadvantage may play a role, as single caregivers are more likely to be living in lower SEIFA areas [43] where more marginalised communities experience a greater proportion of alcohol‐related consequences than more advantaged communities [44, 45]. In the present study, we found that caregivers living in lower SEIFA areas were twice as likely to indicate a child was substantially affected by someone else's drinking than those living in middle to high SEIFA areas. Higher concentrations of alcohol outlets tend to be located in lower socio‐economic areas [46], potentially increasing exposure to alcohol‐related harms [47, 48] or exacerbating existing social, economic and health disparities [35, 49]. Furthermore, caregivers living in more disadvantaged areas may have fewer resources and support systems available to mitigate the negative consequences to their child of others' drinking compared to those in more advantaged areas [43].

### 4.1 Implications

Our findings uniquely provide the perspective of caregivers of children who have been substantially affected by others' drinking and present associations between reported child outcomes and a range of carer socio‐demographic characteristics. This suggests that efforts to address alcohol‐related harms would benefit from simultaneous consideration of social and economic disadvantage. Ensuring availability and accessibility of support (e.g., financial assistance) is crucial as evidence suggests that individuals experiencing barriers such as economic hardship, geographical location constraints and other social factors may be less able to access the support needed [50, 51]. Other measures of support for children and families experiencing alcohol‐related harm might include social assistance such as counselling or peer support groups, and economic assistance, such as subsidies for housing, healthcare or childcare. Temporary experiences of financial stress amongst caregivers with children may be partially addressed with adequate support from state and federal government services, although there is increasing evidence suggesting that the current rate of income support in Australia is insufficient, especially for single caregivers with children [52, 53, 54]. Policies aimed at regulating the density and distribution of alcohol outlets are also needed to address the social and health‐related issues that are associated with higher density of alcohol outlets.

### 4.2 Limitations

Our analysis gives some indication of associations between SDH and children substantially affected by someone else's drinking. However, the analysis is limited by the following constraints and the results should be interpreted cautiously with these limitations in mind. Larger and more targeted studies (i.e., a heavier drinking population or a clinical population) are needed to further examine these relationships. The extent of harms to children from others' drinking is likely underestimated in a general population sample like the one presented here, as individuals in higher‐risk situations may be less likely to participate in survey research [55]. Due to low numbers all predictor variables were dichotomised which may have prevented the examination of meaningful distinctions between categories. Because of small cell sizes and the low statistical power, the analysis was limited to multiple binary logistic regressions instead of multivariable regression models. As a result, possible interactions and the relevance of confounding factors could not be explored and remain a task for future research. Due to the data we had available, our definition of overcrowding was limited to if there were more than two people per bedroom which is not as detailed as the definition outlined by the Australian Institute of Health and Welfare [30]. The outcome used in this study was if a caregiver indicated a child was substantially affected or not, this categorised responses where a child was affected ‘a little’ by others' drinking as ‘not substantially affected’. This is important to consider in interpreting our results as the category ‘not substantially affected’ does not mean that a child was not affected at all by others' drinking, only that the caregivers indicated they were not ‘substantially affected’. Additionally, this measure of harm represents the caregiver's subjective judgement which may vary amongst respondents depending on their own experiences and circumstances. We are unable to identify the relationship between the child and the person whose drinking has substantially affected the child, as these data were not consistently collected across the survey for this item. Finally, our study had a very low response rate where the random digit dialling survey component response rate was 5.5% and the Life in Australia survey was 6.1% [27]. While low response rates for survey research are increasingly common in Australia [56], this may affect the representativeness of our sample and generalisability of our findings. Despite these constraints, our study provides a basis for larger and more targeted studies in this important area.

## 5 Conclusion

We found that select individual, household and area‐based socio‐demographic characteristics were associated with caregivers indicating a child was substantially affected by someone else's drinking. SDH play a considerable role in the health and wellbeing of children, thus policies to address social disadvantage amongst caregivers and children in Australia are needed. Ensuring support services are available and accessible is another factor that needs to be considered by policy makers, as more disadvantaged populations often face social, economic and structural barriers to support services. Future research should include examination of the contexts and environments of children who have been affected by others' drinking, given our findings of significant associations between social and economic disadvantage and caregivers' reports of children being substantially affected.

## Author Contributions

Each author certifies that their contribution to this work meets the standards of the International Committee of Medical Journal Editors.

## Conflicts of Interest

The authors declare no conflicts of interest.
